# Epigenetic and Epitranscriptomic Control in Prostate Cancer

**DOI:** 10.3390/genes13020378

**Published:** 2022-02-18

**Authors:** Judith López, Ana M. Añazco-Guenkova, Óscar Monteagudo-García, Sandra Blanco

**Affiliations:** 1 Centro de Investigación del Cáncer and Instituto de Biología Molecular y Celular del Cáncer, Consejo Superior de Investigaciones Científicas (CSIC)—University of Salamanca, 37007 Salamanca, Spain; judithlopezluis@usal.es (J.L.); ana.anazco@usal.es (A.M.A.-G.); oscar.4mg@gmail.com (Ó.M.-G.); 2 Instituto de Investigación Biomédica de Salamanca (IBSAL), Hospital Universitario de Salamanca, 37007 Salamanca, Spain

**Keywords:** epigenetics, DNA methylation, histone modifications, epitranscriptomics, RNA modifications, prostate cancer, novel therapeutics

## Abstract

The initiation of prostate cancer has been long associated with DNA copy-number alterations, the loss of specific chromosomal regions and gene fusions, and driver mutations, especially those of the Androgen Receptor. Non-mutational events, particularly DNA and RNA epigenetic dysregulation, are emerging as key players in tumorigenesis. In this review we summarize the molecular changes linked to epigenetic and epitranscriptomic dysregulation in prostate cancer and the role that alterations to DNA and RNA modifications play in the initiation and progression of prostate cancer.

## 1. Prostate Cancer

Prostate cancer (PCa) is the second-most diagnosed cancer in men worldwide. In 2019 it accounted for nearly one in five new diagnoses. It is the first cancer in terms of prevalence and is also a leading cause of male cancer-associated deaths [1,2]. Early detection through testing for the prostate specific antigen (PSA) and the improvement of procedures for surgical intervention radiation therapy and androgen deprivation therapy (ADT) have significantly reduced the number of deaths [3]. However, in more advanced or aggressive forms of the pathology, PCa can evolve to stages characterised by invasion of the seminal vesicles followed by metastasis especially in the bone, usually resulting in the death of the patient. The progression to metastatic disease is commonly linked to the fact that the cancer becomes androgen-independent, a frequent feature in advanced prostate cancer [1]. In fact, while ADT is initially effective in the majority of men with PCa, in around 20% of cases, patients progress to castration-resistant prostate cancer (CRPC) for which treatment options are very limited, revealing that other genetic or non-mutational factors may account for the initiation and progression of the disease [4]. Until recently, the first-line treatment options for metastatic CRPC were taxane chemotherapeutic agents [5]; unfortunately, one-third of patients fail to respond to initial treatment and, within 24 months, those who initially respond will develop resistance [6], emphasizing the need to find new therapeutic targets.

Over recent decades, a number of other genetic alterations have been identified associated with PCa. The malignancy is generally characterised by frequent androgen-regulated promoters’ fusion with members of the E26 transformation-specific (ETS) family, such as *ERG*. This fusion was found in 53% of tumours when the complete sequences of seven primary human prostate tumours and their paired normal counterparts were analysed [7]. DNA copy number variations (CNVs) are also frequent [8], especially loss of heterozygosity (LOH), such as a loss of chromosome 8p21 and 10q23 regions, which occurs in up to 85% of high-grade prostatic intraepithelial neoplasias (PIN) and adenocarcinomas [1]. Loss of function alterations in cell cycle genes were found also with high frequency, especially in *CDKN1B* (p27) and *CDKN2A* (p16) genes [9]. Comparative analysis in more advanced and metastatic cancers showed that *APC* alterations were enriched in these types of cancer, while alterations in *ATM* and amplifications in *AR* were specifically enriched in CRPC. Furthermore, other genes such as *PTEN* were commonly found altered in all stages of PCa [10]. Despite all these alterations, no single one was considered as the main driver of the disease.

With the advent of high-throughput next-generation sequencing, recurrent point mutations in several genes, including Androgen Receptor (AR), SPOP, TP53, FOXA1 and PTEN, are found associated with PCa incidence [11]. In recent years, through genomic profiling of prostate tumours with all clinical spectra, high frequencies of somatic and germline alterations have been found in DNA damage repair genes (DDR), phosphatidylinositol 3-kinase (PI3K), and mitogen-activated protein kinase (MAPK) signalling pathways; in genes including *BRCA1*, *BRCA2* and *ATM* (DDR pathway), *PTEN*, *PIK3CA*, *AKT1* (PI3K pathway), *BRAF*, *HRAS* and *KRAS* (MAPK pathway) [10,12], revealing possible drivers of disease initiation, metastasis and castration resistance. 

This emerging era of high-throughput sequencing is revealing a complex scenario of the molecular drivers of prostate cancer, some of which are not associated with permanent DNA changes, but with epigenetic changes such as differential DNA methylation patterns [13]. These findings could improve patient outcomes through the tailored of personalised medicine, and the selection and identification of patient populations with a high risk of developing more aggressive forms of the disease. In the next section, we will comprehensively summarize the role of the epigenetic machinery in regulating the development and progression of prostate cancer, as well as recent advances in the development of epigenetic inhibitors as a promising therapeutic strategy to treat PCa, along with most relevant clinical trials involving epigenetic drugs which are summarised in Table 1.

## 2. Epigenetic Alterations in Prostate Cancer

Until now, profiling studies of primary PCa have been focused on the most studied alterations of this tumour type, such as *AR* alterations, DNA copy number and single point mutations or mRNA expression [29,30,31]. However, with the increase in large-scale genome sequencing and integrated multi-dimensional analyses projects such as The Cancer Genome Atlas (TCGA), the “Encyclopedia of DNA Elements” (ENCODE) or the International Cancer Genome Consortium (ICGC), a different picture started to emerge, where epigenetic changes can lead to chromatin remodelling and aberrant gene expression, which can have severe pathological consequences [32]. In cancer research, recent studies have developed a comprehensive profile of hundreds of primary prostate carcinomas by combining epigenetics, RNA-seq and ChIP-seq [33]. Through multiparametric genomic data integration, it was possible to uncover three subtypes of PCa with differential biological and clinical features, for a tumour type known to be difficult to classify [33]. Other studies have also established PCa subtypes based on distinct epigenetic changes. For instance, in the study by Armenia et al., the authors identified a new class of ETS-fusion-negative PCa defined by epigenetic alterations [34]. Using TCGA methylation and RNA-seq data, Xu et al. performed an epigenetic integrative analysis between normal and PCa tissue, in order to detect the pathways in which DNA methylation-driven genes were significantly enriched [35]. More recently, in the study by Lin et al., using single-cell RNA-seq profiles, the authors identified new signature genes and cell subtypes among CRPC cells [36]. All this evidence brings out a clear role for epigenetic regulation in PCa control.

Mechanistically many studies have shed light on the molecular effects underlying epigenetic dysregulation in PCa. One of the most frequent DNA methylation changes occurs at the *GSTP1* promoter, a fact which was already described 20 years ago. GSTP1 modulates several signalling pathways involved in proliferation, differentiation and apoptosis [37]. After this finding, many other recurrent epigenetic alterations have been described, and may be used in the future as a biomarker for the evaluation of PCa diagnosis and prognosis. Others include the promoter CpG island hypermethylation of *PTEN*, which causes its silencing [38], or the hypermethylation of the tumour suppressor gene *CDKN2A* (which encodes p16) that leads to increased proliferation, thus contributing to carcinogenesis [39]. Even the loss of *AR* expression is regulated in 30% of CRPC by hypermethylation of its promoter [40]. More interestingly, recent studies have described that, in metastatic CRPC and tumours that progress to AR-independency, epigenetic principal regulators are clearly altered, as well as key factor players in chromatin biology [40]. 

Besides DNA methylation, other epigenetic marks regulate chromatin structure and gene expression. Among the plethora of epigenetic master regulators, we can find *writers,* proteins that introduce chemical modifications to DNA and/or histones; *readers* that identify and interpret those modifications with its domains; and *erasers*, whose function is to remove the modifications added by the writers [41]. Uncontrolled activity or expression can lead to tumorigenesis through different molecular events, some of which we highlight here.

### 2.1. Writers

DNA methylation is mainly carried out by a family of DNA methyltransferases (DNMTs). Five different enzymes exist in humans, DNMT1, DNMT3a, DNMT3b, DNMT3c, and DNMT3L. From those, DNMT3a, DNMT3b and DNMT3c directly carry out the de novo methylation, while DNMT3L performs it indirectly [42]. DNMT1 participates in the maintenance of the hemi-methylated DNA strand during replication [43]. DNA methyltransferases are frequently affected epigenetic regulators in PCa, leading to hypermethylation and the subsequent silencing of key tumour suppressor genes or oncogenes including *PTEN*, *CDKN2A*, or *AR* [44,45,46]. Hypermethylation of oncogenes such as *YAP1* has also been associated with neuroendocrine prostate cancer (NEPC) [47]. Expression alterations of DNMT family members have been associated with altered immune infiltration patterns and biochemical recurrence [48]. In addition, the targeted activity of specific methyltransferases can promote tumorigenesis. For example, DNMT1- and DNMT3b-mediated methylation regulates *RAD9* transcription, which induces tumorigenicity [49]. DNMT3a promotes epithelial-to-mesenchymal transition (EMT) by regulating the transcription of key mRNAs [50]. In sum, DNA methylation studies have revealed that DNA methylation levels are a promising approach to classify prostate cancer patients and improve diagnostic tools to predict clinical outcomes more accurately. In addition, drug development efforts to target aberrant DNA hypermethylation led to the development of the DNMT inhibitor 5’-azacitidine. The use of these DNMT inhibitors in pre-clinical studies has been shown to suppress tumour growth [51], and currently, DNMT inhibitors in combination with chemotherapy, AR inhibitors and immunotherapy are in clinical development for prostate cancer (Table 1).

**Figure 1:**
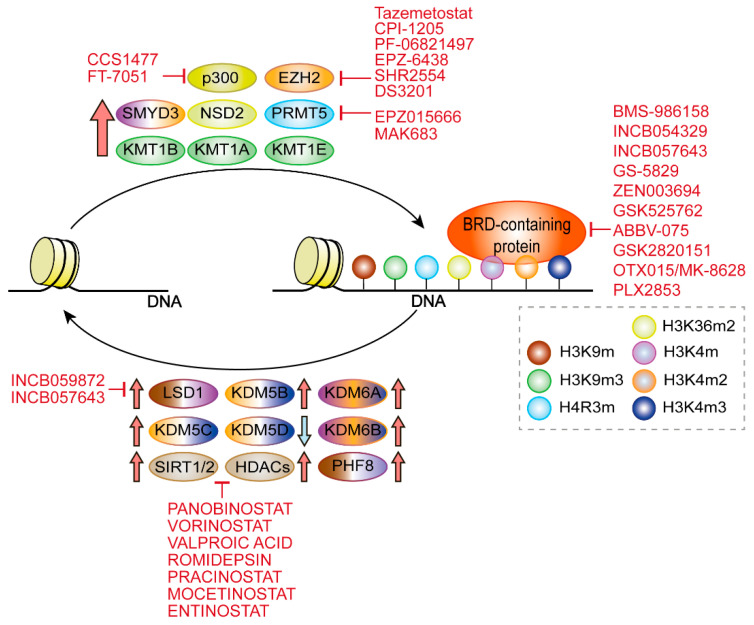
Writers, readers and erasers involved in PCa. Thick arrows indicate over- (red) or under-expression (blue) of the indicated proteins in PCa. Red arrows with flat heads indicate inhibitor compounds under clinical trial against the designated proteins. Writers: KMT1A, KMT1B and KMT1E tri-methylate H3K9; SMYD3 di- and methylates H3K4; PRMT5 methylates H4R3; NSD2 di-methylates H3K36. Erasers: LSD1 demethylates H3K9 and H3K4; KDM5B mono, di- and tri-demethylates H3K4; KDM5B and KDM5C di- and tri-demethylate H3K4. Readers: BRD-containing proteins.

Histone modifications are also common epigenetic marks. The most critical marks are catalysed by histone methyltransferases (HMT) and histone acetyltransferases (HATs). Lysine methyltransferases (KMT) are an important family involved in gene transcriptional regulation [52]. Specifically, KMT1A and KMT1E, also known as SUV39H1 and SETDB1 respectively, are upregulated in PCa cells, increasing their migration and invasion; while KMT1B (or SUV39H2) has been shown to enhance androgen-dependent activity by interacting with AR (Figure 1) [53,54]. Similarly, other epigenetic writers have been proposed as biomarkers for PCa. For instance, the lysine methyltransferase SET and MYND domain-containing protein 3 (SMYD3) is found upregulated in PCa, and its increased expression has been linked to increased cell migration and proliferation (Figure 1) [55]. Upregulated SETDB1 is associated with prognosis and is suggested to promote PCa bone metastases through the WNT pathway [56]. Members of the protein arginine methyltransferase family are also aberrantly expressed in prostate cancer cells, e.g., PRMT5, which catalyses histone arginine methylation at histone H4R3, causing epigenetic inactivation of several tumour suppressors and thus promoting prostate cancer cell growth [57]. 

Alterations in some epigenetic writers such as upregulation of PRMT1 and Coactivator-Associated Arginine Methyltransferase 1 (CARM1) are found in early tumours, suggesting that histone modifications and chromatin remodelling may act as epigenetic drivers at the initial stages of the disease [58]. In addition, within the nuclear receptor binding SET domain (NSD) family, probably the most relevant protein in cancer is NSD2, which was first associated with oncogenesis in tumours such as multiple myeloma [59]; in recent years, it has been found overexpressed in several solid tumours including PCa, where it is especially upregulated in metastatic stages and is correlated with recurrence (Figure 1) [60]. NSD2 catalyses the di-methylation of histone H3 at lysine 36 (H3K36me2), and thus regulating chromatin accessibility and permissive gene transcription [61]. In addition, in vitro studies have indicated that NSD2 modulates TWIST1 to promote EMT and invasiveness in prostate cancer cell lines [62]. More interestingly, NF-kB pathway genes including IL-6, IL-8 or VEGFA are transcriptionally regulated by NSD2, and thus upregulation of NSD2 in CRPC results in enhanced cell proliferation, survival and increased expression of inflammatory cytokines, which in turn induce NSD2 expression through a positive feedback loop [63]. NSD2 also interacts with the AR DNA-binding domain enhancing AR transcriptional activity [64], suggesting that NSD2 may be implicated in promoting ADT tolerance [40]. EZH2, a member of the Polycomb Repressive Complex 2 (PRC2) that regulates transcriptional silencing via histone H3 methylation at lysine 27, is elevated in PCa too [65]. Mechanistically, increased EZH2 expression favours lineage plasticity and neuroendocrine differentiation in androgen-independent tumours [66,67]. EZH2, like other histone modifiers, can also modulate AR recruitment to target sites [68], and EHZ2 inhibition, alone or in combination with AR inhibitors, has resulted in synergistic AR inhibition, resulting in the complete suppression of AR signalling [69,70]. More recently, EZH2 inhibition, by activating the double-stranded RNA-STING stress response, has been shown to increase interferon pathway activity and PD-L1 expression by tumour cells, suggesting that the combination of HMTs inhibitors with immune checkpoint inhibitors could improve the therapeutic outcome [71]. Altogether, these findings have resulted in the initiation of several clinical trials with EZH2 inhibitors, alone or in combination therapy with other targeting agents or immunotherapy (Table 1) (Figure 1).

Histone acetyltransferases (HATs) increased activity can also promote PCa by either acetylating histones or transcription factors, thus inducing transcription [72]. For example, p300 and CREB-binding protein (CBP), by acetylating AR, increase its transcriptional activity [73], and its inhibition in pre-clinical and early clinical trials has shown to downregulate the AR-dependent transcriptional program, modulate the expression levels of several biomarker in CRPC biopsies and tumour growth in both castration-sensitive and castration-resistant prostate tumours [74,75]. More recently, p300/CBP inhibition has been shown to decrease secretion of exosomal PD-L1 by tumour cells, suggesting that the combination of HAT inhibitors with immune checkpoint inhibitors could play a synergic role and improve therapeutic efficacy [76].

All this evidence indicates epigenetic writers as promising therapeutic targets, and several molecules targeting p300/CBP, PRMT5, EZH2 are being tested under clinical trials, alone or in combination with other therapies such as AR blockade or immunotherapy in advanced prostate tumours (Figure 1), which we summarize in Table 1 [16,77,78,79,80]. 

### 2.2. Readers

The bromodomain (BRD)-containing proteins, whose function is to induce transcription initiation through chromatin remodelling, are some of the most studied in prostate cancer cells among other epigenetic readers. More than 70% of NEPC and more than 50% of primary and metastatic PCas show some dysregulation in any of the bromodomain containing proteins [81]. Within this large family, the bromodomain and extraterminal (BET) proteins have been associated with prostate cancer progression. For instance, BRD4, which is the most critical, recognises acetylated lysines at enhancer regions, thus stimulating RNA polymerase II-dependent transcription by recruiting the elongation factor P-TEFb [82]. In addition, in PCa BRD4 also interacts with AR, recruiting AR to sequence-specific DNA-binding motifs to drive AR-mediated gene transcription, making AR-dependent cell lines selectively sensitive to BRD4 inhibitors [83,84]. BET bromodomain proteins have numerous AR-independent functions by activating c-Myc-dependent and c-Myc–independent transcriptional regulators upregulated in CRPC [85].

Apart from the BET family, several BRD-containing proteins have been associated with mCRPC. TRIM24 is a transcription co-regulator overexpressed in CRPC, and is essential for cancer cell proliferation [86]. BAZ2A binds to H3K14ac, repressing transcription and promoting a more aggressive form of PCa [87]. H3K4me2,3 epigenetic reader CHD1 deficiency can lead to a dramatic decrease in cell proliferation, survival, and tumorigenic potential [88]. ATAD2, BRD8, CREBBP, or KTM2A, by recognizing acetylated histones, act as superenhancers and transcriptional regulators that initiate chromatin restructuring to promote tumorigenesis. This evidence show that dysregulated BRD-containing proteins drive aberrant transcriptional programs promoting oncogenesis. This evidence has brought out the potential of targeting these chromatin remodellers, revealing that their inhibition may disrupt the dysregulated transcriptional networks with oncogenic functions. In fact, several small molecule inhibitors, blockers and proteolysis-targeting chimeras (PROTACs), which induce degradation of BET proteins through ubiquitination followed by proteolysis have been developed, and some are currently tested in clinical trials, which we summarize in Table 1 (Figure 1) [16,89,90,91,92].

### 2.3. Erasers

The critical erasers in PCa are DNA demethylases, histone demethylases (HDMT) and histone deacetylases (HDACs), which cause hypomethylation or deacetylation and consequently the upregulation of gene expression, leading to disease progression and higher cell invasion and metastasis [93]. 

To date, the only known DNA demethylases are TET enzymes, comprised of three members, namely TET1-3. DNA demethylation is a three-step process, where TETs participate in the first one, oxidizing m^5^C to 5-hydorxymethylation (hm^5^C) [94]. Decreased expression of TET enzymes has been associated with PCa. For instance, downregulation of TET2 has been implicated in the regulation of AR signalling and prostate cancer development [95,96]. TET1 is downregulated in PCa, and its depletion promotes tumour growth and metastasis in prostate xenograft models and correlates with poor survival rates [97].

Within HDMTs, lysine demethylases (KDM) play important roles in cancer. For example, lysine-specific demethylase 1 (LSD1/KDM1A) overexpression has been highly studied for its oncogenic potential. Its overexpression is associated with increased tumour progression and promotion of carcinogenesis in several cancer types, including PCa [98,99]. In PCa, higher expression of LSD1 is correlated with recurrence and poor survival in metastatic patients, and its function has been shown to be distinctive in androgen-dependent and refractory PCa (Figure 1) [98,100]. In fact, LSD1 is among the best-known modulators of AR transcriptional activity, which can either stimulate or suppress AR transcriptional activity, unveiling a dual role in PCa progression, which is common in other chromatin remodellers [98,101]. In addition, LSD1 can demethylate other key transcription factors in PCa such as FOXA1, leading to its activation and recruitment to AR-dependent enhancers [102]. Moreover, LSD1-mediated epigenetic changes can activate other key genes such as Centrosome-associated protein E (CENPE) [103], as well as cooperate with ZNF217, promoting the expression of genes involved in tumorigenesis, thus leading to CRPC promotion [104].

Many other histone demethylases have been associated to PCa, and similarly to LSD1, their gain or loss of activity seem to have a dual role (Figure 1). H3K4 demethylase KDM5D (JARID1D) is downregulated in metastatic PCa [105], and its loss is associated with resistance to docetaxel in PCa [106]. By contrast, other HMTs are upregulated and their increased activity leads to cancer progression. For instance, KDM5C (JARID1DC) is upregulated in PCa and has recently emerged as a predictive biomarker for therapy failure after prostatectomy [107]. *KDM6A* deletion inhibits tumour progression [108]. KDM6B (JMJD3) regulates c-Myc expression, thus promoting cell proliferation [109]. KDM5B (JARID1DB) plays a role as an AR coactivator, and is upregulated in PCa [110]. Similarly, the KDM4 (JMJD2) family of H3K9 demethylates, either alone or cooperating with LSD1, also play a significant role in modulating AR transcriptional activity, [52,111,112,113], or promoting the expression of AR spliced variants inducing resistance to ADT therapies [114]. KDM4B promotes AR-independent survival by cooperating with BMyb, and its inhibition reduces the growth of AR-independent PCa cells [115]. KDM3B has been implicated in androgen-independent CRPC, and its genetic or pharmacological suppression has shown to reduce the survival of CRPC cells versus castration-sensitive cells [116]. The histone demethylase PHD-finger protein 8 (PHF8) is upregulated in PCa, acts as a transcriptional coactivator of AR via H4K20 demethylation and promotes cancer cell proliferation, migration, invasion, and neuroendocrine differentiation [117,118]. KDM8 (JMJD5) promotes cellular proliferation and controls the activity of AR, HIF-1a, and EZH2 [119]. All this evidence shows that histone methylation dysregulation promotes oncogenesis by promoting chromatin structural changes, and suggests that targeting histone lysine demethylation is a good anti-cancer therapeutic option. In fact, recently developed LSD1 inhibitors have resulted in attenuated tumour growth, and are currently under clinical testing [104,120] (Table 1) (Figure 1). 

Decreased histone deacetylase (HDACs) activity is found associated with PCa too. Contrary to HATs resulting in the increased acetylation of histones or AR, the activity of deacetylases such as SIRT1 or SIRT2 can regulate cellular growth and sensitivity to ADT therapy through AR deacetylation [121], and the use of small molecule inhibitors has been shown to effectively reduce tumour growth, EMT and metastasis, and sensitize mCRPC to ADT therapy (Figure 1) (Table 1) [23,27,122]. These studies have shown that single-agent HDAC inhibitor clinical trials have not shown significant activity. However, their combination with AR inhibitors has resulted in improved therapeutic efficacy [23]. While those results need to be further evaluated with newer AR inhibitors, they suggest that combining HADCs and AR inhibitors may be an effective strategy to re-sensitize tumours to AR inhibition.

In conclusion, all this evidence highlights that epigenetic alterations are commonly associated with PCa and, most importantly, can be used to stratify patient risk, predict clinical outcomes, and to determine best therapeutic options or to reveal molecular pathway vulnerability to distinct chemotherapeutic agents [123,124]. In addition, the oncogenic potential of altered epigenomes in cancer cells suggests that rewiring the epigenome may be an effective strategy to sensitize or kill cancer cells. In fact, several HMT/HDMTs, BET bromodomain, HDACs [40], and DNMT inhibitors are currently under clinical or pre-clinical stages in PCa [125,126], some of which have been approved by the FDA for other malignancies such as myelodysplastic syndrome (MDS), acute myeloid leukaemia (AML) or acute lymphocytic leukaemia (ALL) [127] (Table 1). Yet, further research will be needed to completely understand the extensive transcriptional networks that these epigenetic changes regulate, and how cancer cell fates may be affected. In addition, more studies are necessary to assess their clinical efficacy, to reduce secondary and adverse effects, and to optimise their use in combination with conventional chemotherapies and with predictive biomarkers to select patients that would benefit from these therapies.

## 3. Epitranscriptomics Alterations in Prostate Cancer

Similarly to DNA, RNA can also be modified. Despite being known for over 50 years, the study of RNA modifications has suffered a delay regarding epigenetics, probably due to the lack of suitable tools for their study [128]. Thus, the emergence of this field, known as epitranscriptomics, is closely linked to the recent refinement of tools such as mass spectrometry, next-generation sequencing [128] or cryo-electron microscopy [129], which have enabled the discovery of over 170 RNA modifications [130,131].

These modifications are found in all types of RNA, from messenger RNA (mRNA) to non-coding RNAs such as ribosomal RNA (rRNA), transfer RNA (tRNA), microRNAs (miRNAs) and long noncoding RNAs (lncRNAs) among many others [130]. tRNAs are the more extensively modified, with an average of 15% modified nucleotides per molecule and involving a large number of enzymes and a high diversity of modifications (reviewed in [132,133]). In rRNA, around 130 individual modifications can be found, with 2’-O-methylation of the ribose and pseudouridine (Ψ) being the most frequent modification (reviewed in [134]). In the case of mRNA, the most abundant internal modification is N^6^-Methyladenosine (m^6^A), with around 0.1-0.4% adenines of all mRNAs being modified [135].

In contrast with DNA modifications, which are known to mainly regulate gene expression [136], RNA modifications control many functions apart from transcription such as RNA stability, location, splicing, degradation or translation efficiency [134,137,138]. For instance, 5-methylcytosine (m^5^C) methylation of tRNAs stabilises their structure and protects them from nuclease-mediated cleavage [137]. However, the role or importance of most of these modifications are still unknown and, for others, it is only starting to emerge.

Despite the great diversity of modified nucleotides in RNA and the huge expansion of the field in the past years, little is known about the role of RNA modifications in PCa. In this review, we will summarize the most relevant RNA modifications found to date to have a regulatory role in prostate cancer.

### 3.1. m^6^A Deposition in RNA and Its Role in PCa

**Figure 2:**
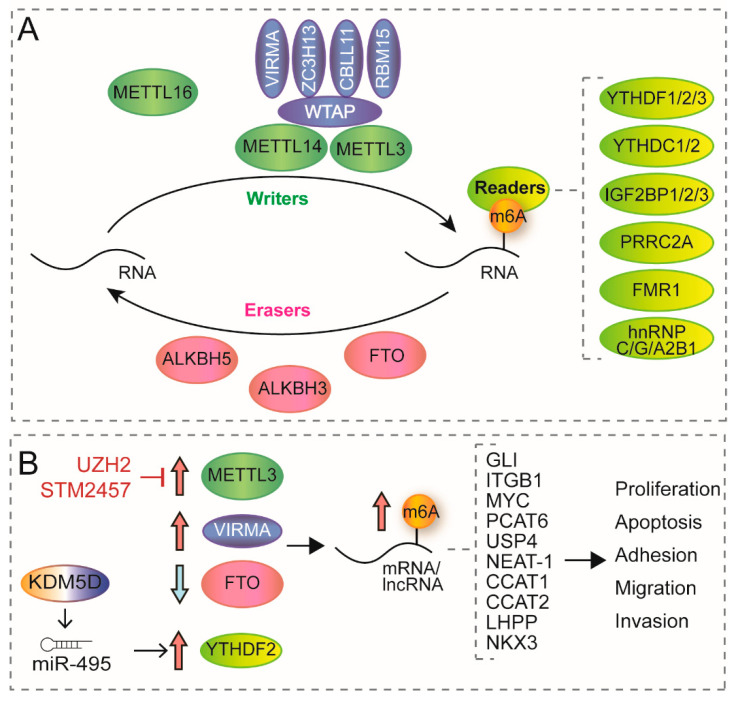
(**A**) Schematic overview of writers, readers and erasers of m^6^A methylation. Green ellipses represent writers. Blue ellipses represent regulators of the writer complex. Red ellipses represent erasers. Yellow ellipses represent readers. Orange circles represent m^6^A methylation mark. (**B**) Relevant m^6^A modifiers in PCa control. Thick arrows indicate over- (red) or under-expression (blue) of the indicated proteins in PCa. Increased or decreased expression of writers, erasers or readers leads to increased m^6^A deposition, which in turn regulates the fate of the indicated mRNAs and lncRNAs, modulating cellular processes: proliferation, apoptosis, adhesion, migration or invasion. Red arrows with flat heads indicate METTL3 inhibitor compounds.

Methylation of position N^6^ of adenosine is the most studied modification. This modification mainly occurs in mRNA, but also in non-coding RNAs such as tRNAs, rRNA, miRNA, lncRNAs and circular RNAs (circRNAs) [139]. Despite the widespread occurrence, m^6^A deposition in mRNA is, by far, the best characterised. Recent improvements in high-throughput methods have demonstrated that m^6^A is not randomly distributed, but is specifically enriched near stop codons in 3’-untranslated regions (UTRs), within long exons, in intergenic regions and introns and at 5’-UTRs [135,139]. 

m^6^A deposition is modulated by the dynamic crosstalk between methyltransferases or “writers” and demethylases or “erasers”. In nascent pre-mRNA, m^6^A is deposited by a multimeric methyltransferase complex. The catalytic core of this complex is constituted by methyltransferase-like 3 (METTL3) and methyltransferase-like 14 (METTL14), which catalyses the formation of m^6^A and recognises RNA substrates, respectively [140]. This complex also requires several cofactors such as RNA-binding motif protein 15 (RBM15), Wilms’ tumour-associated protein (WTAP), Cbl proto-oncogene-like 1 (CBLL1, also known as HAKAI), zinc finger CCCH-type containing 13 (ZC3H13) and Vir-like m^6^A methyltransferase-associated (VIRMA, also known as KIAA1429) [135,140]. Recent studies have identified another m^6^A methyltransferase, METTL16, which acts as an independent RNA methyltransferase and mainly modifies mRNA and small nuclear RNAs (snRNAs) [141]. In addition, METTL5 has also been identified as a 18S rRNA m^6^A methyltransferase acting as a heterodimer together with TRMT112 [142] (Figure 2A). 

Ten years ago, the discovery of two demethylases, fat mass and obesity-associated protein (FTO) and AlkB homolog 5 (ALKBH5), brought to light the reversible nature of this modification [143] and recently, another member of AlkB homolog family, ALKBH3, has been reported as a novel m^6^A demethylase [140]. However, the role of the m^6^A demethylases is still controversial. In 2017, Mauer et al. found that FTO is able to demethylate m^6^Am at the 5’ cap, rather than m^6^A, thereby decreasing mRNA stability. The disparity of this discovery initiates a discussion about m^6^A detection techniques and concerning the nature of its reversibility [144]. Moreover, m^6^A can be specifically recognised by a group of RNA binding enzymes or “readers” that recruit downstream complexes mediating the function of this modification [135]. Several readers have been reported, including: YTH domain-containing proteins (YTHDF1/2/3 and YTHDC1/2), heterogeneous nuclear ribonucleoproteins (including hnRNPC, hnRNPG and hnRNPA2B1), insulin-like growth factor 2 mRNA-binding proteins (IGF2BP1/2/3), proline-rich and coiled-coil-containing protein 2A (PRRC2A) and the fragile X mental retardation 1 (FMR1) [135] (Figure 2A). 

Recent computational studies have found a complex scenario in the expression of several writers, readers and erasers in PCa [145,146,147,148,149], suggesting the use of combined risk scores of several markers for prognosis prediction [145,147,148]. However, other studies have established a clear oncogenic role for increased m^6^A deposition (Figure 2B). For example, METTL3 is overexpressed in PCa patients, leading to increased m^6^A RNA methylation [146,150,151,152,153]. In addition, several studies confirmed that *METTL3* knocked-down (METTL3-KD) represses the proliferation, tumorigenic invasion, and migration capacity of PCa cells in a catalytic-dependent manner in vitro [149,150,151,152,153], and reduces tumour growth in vivo [150]. The downregulation of METTL3-mediated m^6^A methylation leads to the alteration of several downstream pathways. For instance, Cai et al. showed that METTL3 alters proliferation by regulating apoptosis through the Sonic Hedgehog-GLI pathway in a catalytic-dependent manner [150]. MYC proto-oncogene, which plays a key role in PCa, has also been shown to be a target of METTL3 [150,152] and their expression levels correlate in PCa patient tissues [152]. In addition, METTL3 overexpression leads to an increased expression of Integrin B1 (ITGB1) mRNA in a m^6^A-dependent manner, thus altering PCa cell adhesion and motility [151]. Similarly, the lncRNA NEAT-1 is methylated by METTL3 and its expression is higher in bone metastasis [154]. NEAT-1 is recruited to *CYCLIN-L1* and *CDK19* gene promoters through RNA–DNA interactions in a m^6^A-dependent manner, leading to increased proliferation and migration both in vitro and in xenografts [154]. Moreover, METTL3 downregulation affects ubiquitin-specific protease *USP4* mRNA levels, leading to decreased migration and invasiveness in PCa [153]. In addition, one of the pathways by which an increase of METTL3 promotes bone metastasis is through the upregulation of the lncRNA PCAT6 via the m^6^A reader IGFBP2 [155]. These results, together with the increased expression of METTL3 in PCa with bone metastasis compared to primary tumours [151], suggest that METTL3-mediated methylation might play an important role in PCa progression and metastasis. More recently, studies have shown an interplay between m^6^A regulator expression and immune infiltration. In Zhao et al., low METTL3 and HNRNPA2B1 expression correlates with increased immune cell infiltration [156]. In Liu et al. tumours with worse prognosis show a significant decreased expression of METTL14 and ZC3H13, markedly high expression of KIAA1429 and HNRNPA2B1, and are characterised by high intratumor heterogeneity, Th2 cell infiltration, and low Th17 cell infiltration [157], highlighting the functional role of the epitranscriptome in regulating a wide range of oncogenic processes. 

Other members of the methyltransferase complex have been found dysregulated in PCa. VIRMA has been found upregulated in PCa [158], and its knockdown, by regulating the abundance of the oncogenic lncRNAs CCAT1 and CCAT2, can reduce the proliferation, migration and invasion of PCa cells through MYC regulation [158]. The m6A reader YTHDF2 has been found upregulated in PCa, its expression predicts worse overall survival, and its knockdown inhibits proliferation and migration of PCa in vivo and in vitro, by binding and degrading m6A-methylated LHPP and NKX3-1 mRNAs, resulting in increased AKT activity [159]. Interestingly, increased expression of YTHDF2 has been found to be epigenetically regulated by the H3K4me3 demethylase KDM5A. By binding to the miR-495 promoter, KDM5A leads to miR-495 transcriptional inhibition and decreased expression, which results in decreased silencing of *YTHDF2* mRNA [160]. By contrast, FTO has been found downregulated in PCa tissues and cell lines [161]. Similarly to the effects of the increased expression of m^6^A writers, downregulation of FTO leads to increased cell invasion and migration capacity by increasing m^6^A levels [161]. 

In sum, all this evidence suggests that increased m^6^A deposition has an oncogenic effect in prostate cancer cells and targeting METTL3 could have clinical benefits for PCa patients. With the recent emergence of METTL3 as a promising oncogenic target in several haematological malignancies and solid tumours, a great effort is being made to develop small molecule inhibitors to target m6A deposition. Two recent inhibitors have been developed that target and inhibit METTL3, STM2457 and UZH2. These two inhibitors have shown to decrease m^6^A methylation activity in vitro and in vivo. In addition, they have shown potent inhibitory potential in several cancer cell lines including PCa, and a promising anti-cancer effect in cell lines and pre-clinical models of acute myeloid leukaemia (AML) [162,163]. These promising results open the door to explore new therapeutic possibilities in cancer research.

### 3.2. m^5^C and hm^5^C Deposition in RNA and Its Role in PCa

m^5^C deposition in RNA is conserved in all life domains [131] and has been found most prevalently in tRNA and rRNA, but also, less frequently, in mRNA, lncRNA, vault RNA (vtRNA) and small nucleolar RNA (snoRNA) [164,165]. In mammals, there are two families of enzymes known to specifically methylate cytosine at position 5: the NOL1/NOP2/sun (NSUN) family and the DNA methyltransferase family member 2 (DNMT2) [131,165]. To date, NOP2 (also known as nucleolar antigen p120) is the only m^5^C methyltransferase that has been demonstrated to be associated with PCa. Increased expression of NOP2 has been considered a marker of bad prognosis for years, correlating with Gleason score, PSA serum levels and recurrence after radical prostatectomy [166,167]. Mechanistically, NOP2 catalyses the methylation of cytoplasmic 28S rRNA [168]. This methyltransferase is expressed in the late G1 and S phases of the cell cycle and is known to regulate nuclear activation associated with proliferation [166]. In addition, a recent genome-wide association study combining enhancer and eQTL mapping has reported that lower expression of NSUN4 is associated with an increased prostate cancer risk [169]. NSUN4 targets 12S mitochondrial rRNA, found on the small subunit [170], yet its potential role in PCa remains to be validated. In addition, whether other m^5^C RNA methyltransferases are associated with PCa is also still uncovered.

Similarly to DNA, hm^5^C and f^5^C have been found in mRNA and tRNAs in vivo [171]. ALKBH1 has been found to catalyse the conversion of m^5^C into f^5^C in tRNAs in vivo [172]. In vitro studies have shown that TET enzymes are able to oxidise m^5^C to hm^5^C in RNA [173]. Despite this finding, the enzyme that catalyses hm5C conversion in mRNA is still unknown. TET2 is found downregulated in PCa [95,174], yet whether decreased hm5C RNA levels are linked to PCa needs to be elucidated.

From a therapeutic point of view, m^5^C as well as other RNA modifications have been linked to the survival, proliferation and differentiation of tumour cells in several cancers [175,176,177]. Interestingly, m^5^C as well as other RNA modifications have been associated with drug resistance regulation [175,177,178]. For instance, combined knockdown of *NSUN2* and *METTL1*, which catalyses tRNA 7-methylguanosine, has shown to sensitize tumour cells to the chemotherapeutic agent 5’-fluorouracil (5-FU) [178], confirming the link between RNA methylation, cell survival and chemotherapy. In addition, in skin cancer, a lack of NSUN2 specifically sensitizes tumour-initiating cells to 5-FU [177], further showing that the combination of classic chemotherapeutic agents and the inhibition of RNA modifications could become a more effective therapeutic strategy. This role of RNA modifications has not been evaluated in PCa, but aforementioned investigations linking several RNA-modifying enzymes with key PCa tumorigenic pathways will pave the way to finding novel therapeutic targets.

### 3.3. Pseudouridine in RNA and Its Role in PCa

Pseudouridine (Ψ), the C5-glycoside isomer of uridine, is among the most well-known RNA modifications. It comprises about 5% of the total of all RNA nucleotides and is found in almost all types of non-coding and coding RNAs [179]. Ψ biosynthesis can be carried out both by stand-alone enzymes and by RNA-guided ribonucleoprotein (RNP) complexes. To date, eleven pseudouridine synthases have been identified in humans; PUS1, TRUB2, PUS3, PUS4, RPUSD1, RPUSD2, RPUSD3, RPUSD4, PUS7, PUS7L, and PUS10 [180]. RNP complexes are constituted by the enzymatic core components dyskerin (DKC1), which carries the enzymatic activity, and three additional core proteins: nucleolar protein 10 (NOP10), glycine-arginine-rich protein 1 (GAR1) and non-histone protein 2 (NHP2); in addition, non-coding RNAs called H/ACA box snoRNAs that guide the protein complex to the specific uridines to be modified and NAF1 and SHQ1, which are H/ACA-specific chaperones [181]. This modification is irreversible and has shown to play an important role in gene expression regulation and maintenance of structural stability [135]. Similarly, the fate of Ψ-targeted RNAs is dependent on their readers. Recent studies have identified MetRS as a potential Ψ reader [182]. 

Aberrant deposition of Ψ, dysregulated expression of pseudouridylases and somatic mutations have been linked to PCa. For instance, in an integrative genomic profiling study of prostate cancer, somatic mutations in the chromosomal 3p were identified as tumour suppressors; most importantly, *SHQ1* was the only gene in the 3p region that harboured tumour associated mutations [7,30], and loss-of-function studies confirmed a tumour growth-suppressive role of SHQ1 [183]. DKC1 is overexpressed in PCa and its knockdown decreases cell proliferation in prostate adenocarcinoma cell lines [184]. While, mechanistically, it is still unknown whether increased DKC1 expression correlates with Ψ increased levels, some studies have shown that targeting its binding site to the RNA component of Telomerase (hTR) can modulate telomerase activity [185]. However, Ψ levels have been found to be increased in the rRNA of PCa cell lines compared to prostate epithelial cells, as well as in PCa tissue compared to normal adjacent tissues [186], suggesting an oncogenic role for elevated Ψ levels. Computational analysis has also shown the upregulation of several H/ACA snoRNAs and amplification of *DKC1* in metastatic tumours compared to primary tumours [186,187,188,189]. Furthermore, the studies showed that the silencing of those snoRNA in PCa cell lines reduced their proliferation and metastatic potential [189,190]. 

Other functional studies have highlighted an oncogenic role for increased Ψ deposition. For example, the stand-alone pseudouridine synthase PUS10 has shown to play a key role in TRAIL-induced apoptosis, participating in the release of cytochrome C and SMAC from the mitochondria in PCa cell lines [191]. Moreover, increased abundance of PUS1 was recently found to be associated with an increased risk of relapse [192]. Furthermore, metabolomic analysis of urine samples has found increased levels of Ψ in PCa patients, suggesting Ψ levels in liquid non-invasive biopsies could be a novel predictive biomarker to use in combination with others, such as PSA levels, in order to improve diagnosis [193]. Taken together, the evidence so far shows that Ψ increased deposition has an oncogenic potential, yet the molecular mechanisms underlying this effect still need to be fully investigated to establish whether targeting Ψ writers may have clinical benefits for PCa patients. Nonetheless, Ψ arises as a promising minimally invasive biomarker for PCa detection, but further studies and standardization are needed in order to obtain a fast, cost-effective detection method for the clinical practice. 

### 3.4. RNA Editing in PCa

RNA editing is also a very prevalent post-transcriptional event in human transcriptomes. The most common type of RNA editing results from the conversion of adenosine (A) to inosine (I) in double-stranded RNA, a process catalysed by the adenosine deaminase acting on the RNA (ADAR) family of enzymes. A-to-I editing is highly prevalent within Alu elements, as well as introns, untranslated regions (UTRs) and coding transcripts [194]. In a comprehensive study to identify edited RNAs in prostate cancer patients, 16 paired DNA–RNA sequence libraries from prostate tumour specimens were analysed. Over a hundred thousand putative RNA editing events were found on introns and UTRs, and coding regions predicted to result in deleterious amino acid alterations [195]. In a later transcriptome-wide study, elevated RNA-editing and expression of ADAR enzymes were found across multiple cancer tissues, including the prostate [196]. Moreover, rare germline heterozygous variants in ADAR predispose to prostate cancer [197]. Despite these findings, little is known on the mechanistic link between elevated RNA editing levels and PCa, and few have explored the molecular causes. For instance, RNA editing of AR mRNA has been found in PCa cell lines, which leads to mutated AR with a gain-of-function activity, suggesting a contribution to hormone-refractory phenotypes [198]. Another study implicates an inflammation-driven malignant transformation due to increased type I interferon expression [197]. 

Taken together, these studies disclose an important regulatory mechanism in cancer for RNA editing; however, a systematic effort to define the putative roles of edited RNAs in PCa tumorigenesis and progression remains to be established.

### 3.5. 2′-O-methylation in PCa

One of the most abundant ribonucleotides in rRNA is 2′-O-methylation (Nm, ribomethylation), with up to 112 different positions in human ribosomes. Similarly to Ψ, its deposition is carried out by RNA-guided ribonucleoprotein (RNP) complexes formed by the core proteins NOP56, NOP58, SNU13 and fibrillarin (FBL) and the guiding box C/D snoRNAs [199]. Altered expression or mutations in the components of the 2′-O-methylation machinery have been found to be associated with several cancer types, yet in PCa, only expression changes in snoRNA U50 have been associated with a tumour suppressor function in PCa [200]. More recently, a study unveiled a methyltransferase-independent function of the oncogene EZH2 that relies on a direct interaction with fibrillarin, leading to enhanced rRNA 2′-O methylation and protein translation [201]. This study highlights an important alliance between epitranscriptomic and epigenetic pathways in tumorigenesis, and suggests that increased 2′-O methylation may have important consequences for cancer development.

## 4. Concluding Remarks

Despite the initial response to hormone-deprivation treatment, one of the main problems in PCa management is the relapse and progression rate to metastatic tumour, which has limited therapeutic options, none of them completely curative [40,202]. This intensifies the urgent need for the investigation of new therapeutic approaches.

Recent evidence has highlighted that epigenetic alterations are emerging as potential biomarkers to stratify PCa patients and predict clinical outcomes [40]. Epigenetic alterations are most common in advanced PCa, being especially dysregulated in metastatic CRPC [13]. These findings suggest an important role of epigenetic regulation in advanced phases of the disease and indicate that epigenetic mechanisms may regulate tumour selective pressures. The use of epigenetic modulators has been growing in recent years, and currently, six epigenetic drugs are approved by FDA for cancer treatment, mainly for haematological malignancies [127]. Regarding PCa, despite the huge number of studies pointing to epigenetic modulators as prognostic markers, none of them are used nowadays in clinical practice. However, clinical trials have shown only mild results in PCa patients, probably because most of them have been undertaken in late-stage, heavily pre-treated patients and without considering tumour subtypes [127,203,204]. A deep understanding of the molecular mechanism underlying the epigenetic mechanism and tumour biology will allow the development of successful clinical trials and the eventual approval of epigenetics-based therapies for PCa.

As in DNA, RNA modifications are also known to regulate responses to environmental signals [137,177] suggesting that they too may regulate cancer cells’ survival of challenges occurred during tumour expansion or therapies, making them attractive therapeutic targets. However, unlike epigenetics, epitranscriptomics has not reached the clinic yet. Changes in several RNA modifications have been linked to different tumours including PCa [150,151,152,154,158,160,180,186,204], revealing their potential role as tumour biomarkers. However, their use is still limited by the lack of easy, sensitive, cost-effective and reliable high-throughput detection methods. In addition, the aberrant expression of RNA-modifying enzymes has also been reported in PCa [150,151,152,158,166], but their specific roles in regulating tumorigenesis remain to be further characterised.

Similarly to epigenetics, RNA modifications are emerging as promising therapeutic targets, and great efforts are now being made to develop small molecule inhibitors to rewire the aberrant cancer epitranscriptomes. However, targeting RNA modifications could be fairly complicated since they are linked to most aspects of RNA biology, and their alteration could involve undesirable toxic effects. Moreover, the role of RNA modifications is context-dependent and could differ between cancers or even between different cell populations [177,205]. Thus, there is still a long road ahead that will require great research efforts in order to fully understand the biology of RNA modifications and the means to effectively target them, so that ground-breaking epitranscriptomics can finally reach the clinic. 

## Figures and Tables

**Table 1:** Epigenetic inhibitors in clinical trials for PCa.

DRUG	TRAIL ID	PHASE	PROTOCOL	STATUS
**DNMT INHIBITORS**				
5-AZACYTIDINE	NCT00384839	Phase II	Patients with CRPC received 75 mg/m^2^ of 5-azacytidine for five consecutive days of a 28-day cycle. Patients were treated until clinical progression up to a maximum of 12 cycles.	Completed. 5-Azacytidine modulates PSA (doubling time > 3 months) in 56% of patients. Clinical progression-free survival of 12.4 weeks [14]
5-AZACYTIDINE	NCT00503984	Phase I/II	mCRPC (+docetaxel, prednisone)	Completed [15].
5-AZACYTIDINE	NCT00006019	Phase II	mCRPC (+ Sodium phenylbutyrate)	Completed.
DISULFIRAM	NCT01118741		CRPC	Completed.
DECITABINE	NCT03709550	Phase I/II	mCRPC (+Enzalutamide)	Not yet recruiting.
5-AZACYTIDINE	NCT02959437	Phase I/II	Advanced Solid tumours (+ PD-1 + IDO-1)	Terminated by Sponsor
**HMT INHIBITORS**				
PRMT5 INHIBITOR MAK683	NCT02900651	Phase I/II	Diffuse large B cell lymphoma, advanced solid tumours	Recruiting
EZH2 INHIBITOR TAZEMETOSTAT	NCT03213665	Phase II	Advanced solid tumours	Active. Not Recruiting
EZH2 INHIBITOR CPI-1205	NCT03480646	Phase I/II	mCRPC (+Abiraterone/prednisone or enzalutamide)	Active. Not Recruiting
EZH2 INHIBITOR PF-06821497	NCT03460977	Phase I	mCRPC	Recruiting
EZH2 INHIBITOR EPZ-6438	NCT04179864	Phase Ib	mCRPC (+Abiraterone/prednisone or enzalutamide)	Recruiting
EZH2 INHIBITOR SHR2554	NCT03741712	Phase I/II	mCRPC (+SHR3680)	Terminated
EZH1/2 INHIBITOR DS3201	NCT04388852	Phase I/II	mCRPC (+Ipilimumab)	Recruiting
**HAT INHIBITORS**				
P300/CBP INHIBITOR CCS1477	NCT03568656	Phase I/II	mCRPC (+Abiraterone/prednisone or enzalutamide)	Recruiting
P300/CBP INHIBITOR: FT-7051	NCT04575766	Phase I	mCRPC	Recruiting
**BRD-CONTAINING PROTEIN INHIBITORS**				
BMS-986158	NCT02419417	Phase I/II	Advanced solid tumours	Active. Not Recruiting
INCB054329	NCT02431260	Phase I/II	Advanced solid tumours	Terminated
INCB057643	NCT02711137	Phase I/II	Advanced solid tumours (+abiraterone)	Terminated
GS-5829	NCT02607228	Phase I/II	mCRPC (+enzalutamide)	Terminated
ZEN003694	NCT02711956	Phase I/II	mCRPC (+enzalutamide)	Completed. Longer PFS in a subset of patients [16].
ZEN003694	NCT02705469	Phase I	mCRPC	Completed.
ZEN003694	NCT04471974	Phase II	mCRPC (+Enzalutamide + pembrolizumab)	Recruiting
GSK525762	NCT03150056	Phase I	mCRPC (+Abiraterone/prednisone or enzalutamide)	Completed
ABBV-075	NCT02391480	Phase I	mCRPC	Completed. Not significant antitumour activity [17].
GSK2820151	NCT02630251	Phase I	Advanced or recurrent solid tumours	Terminated (In 2017, GSK2820151 was terminated due to development of another BET Inhibitor (GSK525762) with a better understanding of the risk benefit profile.)
OTX015/MK-8628	NCT02259114	Phase Ib	mCPRC	Completed. Not significant antitumour activity [18].
PLX2853	NCT04556617	Phase I/II	mCPRC (+Abiraterone/prednisone or olaparib)	Recruiting
**HDMT INHIBITORS**				
LSD1 INHIBITOR: INCB059872	NCT02712905	Phase I/II	Solid tumours and hematologic malignancy	Active. Not Recruiting
LSD1 INHIBITOR: INCB059872	NCT02959437	Phase I/II	Advanced Solid tumours (+pembrolizumab + epacadostat)	Terminated by Sponsor
LSD1 INHIBITOR: INCB057643	NCT02959437	Phase I/II	Advanced Solid tumours (+pembrolizumab + epacadostat)	Terminated by Sponsor
**HDAC INHIBITORS**				
VORINOSTAT/SAHA	NCT00005634	Phase I	mCRPC	Completed. Determine the tolerability, pharmacokinetic profile, and biological effects of the drug. Not available [19].
VORINOSTAT/SAHA	NCT00330161	Phase II	mCRPC with disease progression on prior chemotherapy received 400 mg vorinostat/SAHA orally each day. Disease progression measured at 6 months. n = 27	Completed. Toxicity: significant toxicities including fatigue, nausea. IL-6 was higher in patients with toxicity. Seven percent of patients achieved a stable disease state. No PSA decline >50% observed. Median time to progression and overall survival were 2.8 and 11.7 months, respectively. Significant toxicities reported [19].
VORINOSTAT/SAHA AND DOCETAXEL	NCT00565227	Phase I	Patients with advanced and relapsed tumours received oral vorinostat/SAHA for the first 14 days of a 21-day cycle, with docetaxel I.v. on day 4 of each cycle. n = 12	Completed. Toxicity: neutropenia, peripheral neuropathy, and gastrointestinal bleeding. The combination of vorinostat/SAHA and docetaxel was poorly tolerated. No responses were identified [20].
VORINOSTAT/SAHA	NCT00589472	Phase II	Localised PCa (+Bicalutamide, goserelin acetate, or leuprolide acetate)	Completed.
VALPROIC ACID	NCT00530907	Phase I	CRPC (+Bevacizumab)	Completed [21].
PANOBINOSTAT (LBH589)	NCT00667862	Phase II	I.v. panobinostat (20 mg/m^2^) was administered to CRPC patients on days 1 and 8 of a 21-day cycle. Disease progression measured at 24 weeks. n = 35	Completed. Toxicity: fatigue, thrombocytopenia, nausea; 14% of patients demonstrated a decrease in PSA but none >50%. No clinical activity [22].
PANOBINOSTAT	NCT00878436	Phase I/II	CRPC (+bicalutamide)	Completed [23].
PANOBINOSTAT, DOCETAXEL, AND PREDNISONE	NCT00663832	Phase I	CRPC patients received oral panobinostat (20 mg/m^2^) the first, third and fifth day of the week for 2 consecutive weeks. In addition, patients received oral Panobinostat (15 mg/m^2^) with docetaxel I.v. (75 mg/m^2^) every 21 days and oral prednisone (5 mg) twice every day of a 21-day cycle. n = 16	Completed. Toxicity: dyspnoea and neutropenia The combination in patients with CRPC resulted in 63% of patients with >50% decline in PSA levels. No relevant anti-tumour activity [24].
PANOBINOSTAT DOCETAXEL AND PREDNISONE	NCT00493766	Phase I	On the one hand, oral panobinostat alone is given to patients with progressing hormone refractory prostate cancer. On the other hand, oral Panobinostat along with I.v. docetaxel and oral prednisone is administered. n = 16	Completed. Toxicity: dyspnoea, neutropenia, fatigue. Exposure to oral panobinostat was similar with and without docetaxel [25].
PANOBINOSTAT	NCT00670553	Phase I	Localised prostate cancer (+External beam radiotherapy)	Completed.
PANOBINOSTAT	NCT00667862	Phase I	mCRPC	Completed.
ROMIDEPSIN	NCT00106418	Phase II	mCRPC patients received romidepsin (13 mg/m^2^) intravenously on days 1, 8, and 15 every 21-day cycle. Disease progression measures at 6 months. n = 35	Completed. Toxicity: nausea, fatigue. Two patients reached a confirmed radiological partial response of over 6 months, in addition to >50% PSA decline. Eleven patients had to discontinue the therapy due to toxicity. Romidepsin demonstrated minimal anti-tumour activity in chemonaive patients with CRPC [26]
PRACINOSTAT (SB939)	NCT01075308	Phase II	mCRPC	Completed [27].
MOCETINOSTAT (MGCD0103)	NCT00511576	Phase I	Advanced cancer tumours	Celgene terminated its collaboration agreement with MethylGene for the development of MGCD0103.
ENTINOSTAT (MS-275)	NCT03829930	Phase I	CRPC (+Enzalutamide)	Terminated (Sponsor discontinued the drug).
ENTINOSTAT (MS-275)	NCT00020579	Phase I	Advanced solid tumours or lymphoma.	Completed [28].
